# Disease severity of children with hereditary spherocytosis predicts the clinical course of aplastic crisis

**DOI:** 10.1007/s00431-026-07211-y

**Published:** 2026-07-06

**Authors:** Shiri Rubin, Shira Devora Ben Ami, Ohad Atia, Dafna Brik Simon, Tracie Alison Goldberg, Michal Dvori, Galit Pinto Berger, Merav Shalit, Tamar Feuerstein, Oded Gilad, Joanne Yacobovich, Orna Steinberg-Shemer

**Affiliations:** 1 Department of Hematology-Oncology, Schneider Children’s Medical Center of Israel, 14 Kaplan St., 4920235 Petah Tikva, Israel; 2 Gray Faculty of Medical and Health Sciences, Tel Aviv University, 35 Klatzkin St., 6997801 Tel Aviv-Yafo, Israel; 3 Present Address: Department of Pediatric Hemato-Oncology, Dana Dwek Children, Tel Aviv Sourasky Medical Center, Ichilov, 6 Weizmann St, 64239 Tel Aviv-Yafo, Israel

**Keywords:** Hematology, Hereditary spherocytosis, Aplastic crisis, Parvovirus B19, Pancytopenia, Hemolytic anemia

## Abstract

Transient aplastic crisis (TAC) is a known complication of hereditary spherocytosis (HS), typically triggered by parvovirus-B19. However, in children with HS, the occurrence, clinical course and predictors of TAC severity remain incompletely defined. We aimed to characterize the clinical and hematologic features of TAC and to identify factors associated with its severity. We conducted a single-center retrospective cohort study of children with HS, analyzing clinical presentation, laboratory parameters, and outcomes. Associations between baseline disease severity and TAC course were assessed using correlation analyses and multivariable logistic regression. We studied 123 children with HS of whom 61 (49.6%) experienced TAC (median age 6.6 years, interquartile range (IQR) 4.56–8.18). Pancytopenia was observed in 28 (50.9%) of 55 patients with complete data and was associated with a lower mean nadir hemoglobin level (5.3 ± 1.0 vs. 5.9 ± 0.8g/dL, *p *= 0.009) and a higher transfusion requirement (median 2, IQR 1.8–2 vs. 1, IQR 1–2 units, *p* < 0.001). Acute parvovirus-B19 infection was confirmed in 94.4% of those tested. Lower baseline hemoglobin (*ρ* = − 0.26, *p* = 0.039) and higher baseline reticulocyte percentage (*ρ* = 0.52, *p* < 0.001) correlated with greater transfusion requirements. Baseline HS characteristics were more severe in patients who developed pancytopenia during TAC. In multivariable analysis, higher baseline reticulocyte percentage and older age at crisis were independently associated with greater TAC severity. All the patients achieved complete hematologic recovery.

*Conclusion*: TAC-related pancytopenia is common in children with HS. Baseline hemolytic severity is associated with a more severe clinical course of TAC, supporting risk-adapted monitoring and management during aplastic crisis.

**Table:** 

What is Known: • *Transient aplastic crisis (TAC) is a recognized complication of hereditary spherocytosis, most commonly triggered by parvovirus-B19.* • *TAC is classically described as pure red cell aplasia but may be accompanied by additional cytopenias.*
What is New: • *In the largest pediatric cohort to date, pancytopenia occurred in about half of TAC events and was associated with increased transfusion requirements.* • *Baseline hemolytic severity predicted the clinical course of TAC, including pancytopenia and transfusion requirements, supporting risk-adapted monitoring.*

What is Known:

• *Transient aplastic crisis (TAC) is a recognized complication of hereditary spherocytosis, most commonly triggered by parvovirus-B19.*

• *TAC is classically described as pure red cell aplasia but may be accompanied by additional cytopenias.*

What is New:

• *In the largest pediatric cohort to date, pancytopenia occurred in about half of TAC events and was associated with increased transfusion requirements.*

• *Baseline hemolytic severity predicted the clinical course of TAC, including pancytopenia and transfusion requirements, supporting risk-adapted monitoring.*

## Introduction

Hereditary spherocytosis (HS), the most common inherited red blood cell (RBC) membrane disorder, is caused by defects in proteins anchoring the cytoskeleton to the lipid bilayer. This leads to the production of spherical, poorly deformable erythrocytes prone to hemolysis [1]. HS is characterized by chronic hemolytic anemia, jaundice, and splenomegaly, with a heterogeneous clinical severity [2, 3]. Diagnosis is based on clinical and laboratory findings and confirmed by the osmotic fragility test or eosin-5-maleimide (EMA) binding by flow-cytometry [4–6]. Management includes folic acid supplementation, RBC transfusions, and splenectomy in severe cases [7].

A major complication of HS is transient aplastic crisis (TAC), characterized by acute suppression of erythropoiesis with severe anemia and reticulocytopenia. TAC may be the initial clinical manifestation of previously undiagnosed HS, particularly in children with a mild course of HS [8–12]. TAC is most commonly triggered by parvovirus-B19 infection [13], a DNA virus that selectively infects progenitor cells in the bone marrow (BM). Laboratory diagnosis of acute parvovirus-B19 infection relies on serologic detection of IgM antibodies or molecular detection by polymerase chain reaction (PCR); combined testing achieves the best diagnostic accuracy [13–15]. While infection is usually mild in immunocompetent individuals, chronic hemolytic anemias, such as HS, increase vulnerability due to the shortened RBC lifespan [13, 16].

Several case reports and small case series of parvovirus-B19-associated TAC described concomitant leukopenia and thrombocytopenia, and sometimes pancytopenia, in healthy individuals and in patients with underlying hemolytic anemia [12, 17–20]. Various mechanisms have been proposed to explain pancytopenia associated with parvovirus-B19 infection, including direct viral toxicity mediated by non-structural proteins (e.g., NS-1), viral binding to hematopoietic progenitor cells via the P-antigen, and immune-mediated processes such as virus-associated hemophagocytic syndrome [21]. Hanada et al*.* described a child with HS and pancytopenia whose clinical course improved following administration of intravenous immunoglobulin; in-vitro studies demonstrated reversible inhibition of myeloid and megakaryocyte differentiation by the patient’s serum during the acute phase [22]. These observations suggest that parvovirus-B19 may induce a broader suppression of hematopoiesis beyond the erythroid lineage.

This study represents the largest systematic evaluation of pediatric patients with HS experiencing TAC. We found that lower baseline hemoglobin and higher reticulocyte percentage were related to the occurrence and severity of pancytopenia and of transfusion requirements during TAC.

## Methods

### Patients and data extraction

We conducted a single-center retrospective cohort study of all the patients with HS followed at the Schneider Children’s Medical Center during 1995–2025. Data extracted from patient’s charts included family history, clinical and laboratory data, and HS-related complications. Baseline hemoglobin level, reticulocyte percentage, and absolute reticulocyte count (ARC) were each defined as the mean of all the measurements obtained between diagnosis and TAC onset. Similarly, the number of hospitalizations and RBC transfusions was calculated between diagnosis and TAC onset among patients who experienced TAC. Patients with insufficient clinical information were excluded. The study was approved by the Institutional Review Board of Rabin Medical Center.

### Diagnosis of hereditary spherocytosis

HS diagnosis was based on family history, clinical and laboratory data, RBC morphology, and the diagnostic assays, including the osmotic fragility test and the EMA-binding test; the latter was incorporated since 2014. The osmotic fragility test was performed as previously described [23]. In short, fresh and 24-h incubated samples were exposed to gradually reduced NaCl concentration (0.85%−0%) to generate hemolysis curves. Increased fragility was interpreted relative to the Wintrobe reference ranges. For the EMA-binding test, washed RBCs were incubated with 0.5mg/mL EMA for 1 h, washed with PBS, and analyzed by flow cytometry. The results were expressed as the percent decrease in mean fluorescence intensity relative to the control samples. A decrease of > 21% was considered diagnostic for HS [24].

### Definitions of transient aplastic crisis and pancytopenia

Aplastic crisis was defined as an acute episode of severe anemia and reticulocytopenia relative to baseline levels. Pancytopenia was defined as anemia accompanied by a white blood cell (WBC) count < 4,000/µL and a platelet count < 150,000/µL. Pancytopenia was also evaluated using low absolute neutrophil count (ANC); however, as the results were similar, the WBC-based definition was used for all the analyses.

### Parvovirus-B19 detection

Acute parvovirus-B19 infection was defined by the presence of specific IgM antibodies, assessed using enzyme-linked immunosorbent assays (ELISA) or PCR detection of viral DNA in blood [25, 26].

### Statistical analysis

Statistical analyses were performed using Microsoft Excel (version 16.103.3; Microsoft Corporation, Redmond, WA), GraphPad QuickCalcs (GraphPad, San Diego, CA), and Python (version 3.8) utilizing the NumPy library and the SciPy statistical module (scipy.stats).

Categorical variables were compared using the χ^2^ test or Fisher’s exact test, as appropriate, based on the expected frequencies in the contingency tables. Continuous variables were assessed for normality; variables with normal distributions were summarized as mean ± standard deviation (SD) and compared using unpaired t-tests. Non-normally distributed variables were summarized as median with interquartile range (IQR) and compared using the Mann–Whitney U test. Correlations between normally distributed continuous variables were assessed using Pearson’s correlation coefficient, while correlations between non-normally distributed continuous variables were assessed using Spearman’s rank correlation coefficient.

Multivariable logistic regression models were used to identify independent predictors of pancytopenia and transfusion burden during TAC. Baseline hemoglobin level, baseline reticulocyte percentage, and prior transfusion history were included as candidate predictors, while age at crisis and sex were incorporated as adjustment covariates. An interaction within the model between baseline hemoglobin and baseline reticulocyte percentage was tested but not retained due to lack of statistical significance.

All the statistical tests were two-sided, and a *p*-value < 0.05 was considered statistically significant.

## Results

### Characteristics of patients with HS

Of 123 patients with HS, 70 (56.9%) were males. The median age at diagnosis was 0.2 years (IQR 0.04–1.99); 80 (65.0%) were diagnosed in the first year of life. Overall, 104 patients (84.6%) were diagnosed before age 4 years. A family history of HS was documented in 100 patients (81.3%). The mean baseline hemoglobin level was 9.8 ± 1.4 g/dL, the mean reticulocyte percentage was 7.9 ± 2.9%, and the mean ARC was 290.5 ± 122.9 × 10^3^/µL.

The median total bilirubin level was 2mg/dL (IQR 1.5–3). Splenomegaly was reported in 91.9%. Patients experienced a median of 1 (IQR 0–3) hospitalization and received a median of 2 (IQR 0–5.5) RBC transfusions. Splenectomy was performed in 36 (29.3%) and cholecystectomy in 51 (41.5%) (Table 1).

**Table 1:** Patient characteristics

	*n* = 123 (%)
Sex	
Male	70 (56.9%)
Female	53 (43.1%)
Age at diagnosis, years median (IQR)	0.2 (0.04–1.99)
Family history	100 (81.3%)
Laboratory findings at baseline	
Hemoglobin (g/dL), mean ± SD	9.8 ± 1.4
Reticulocytes (%), mean ± SD	7.9 ± 2.9
Absolute reticulocyte count (× 10^3^/µL), mean ± SD	290.5 ± 122.9
Total bilirubin (mg/dL), median (IQR)	2 (1.5–3)
Splenomegaly	113 (91.9%)
Number of hospitalizations, median (IQR)	1 (0–3)
Number of RBC transfusions, median (IQR)	2 (0–5.5)
Splenectomy	36 (29.3%)
Cholecystectomy	51 (41.5%)

*IQR* interquartile range, *SD* standard deviation, *RBC* red blood cell

### Clinical presentation of TAC

Sixty-one patients (49.6%) experienced TAC. The median age at crisis was 6.6 years (IQR 4.6–8.2). For three, TAC was the initial clinical presentation, leading to the subsequent diagnosis of HS. Importantly, TAC was not observed following splenectomy in any patient with HS. Among the 36 patients who underwent splenectomy, 19 (52.8%) experienced TAC prior to splenectomy, and 17 (47.2%) were never diagnosed with TAC. Among the 51 patients who underwent cholecystectomy, 3 (5.9%) underwent the procedure before TAC, 23 (45.1%) after TAC, and 25 (49.0%) never experienced TAC.

**Fig. 1:**
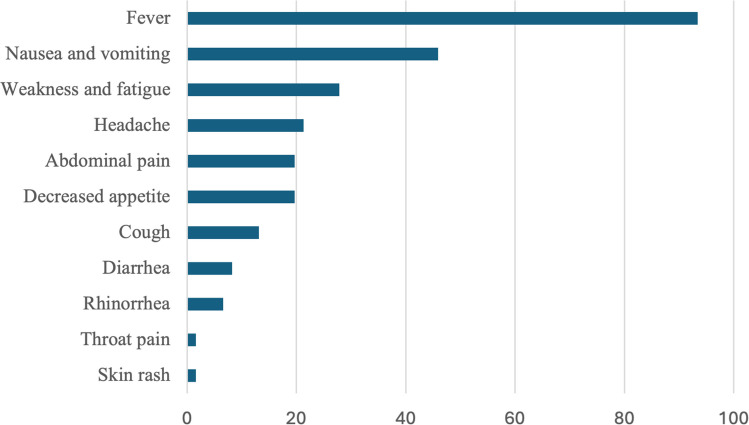
Clinical presentation of transient aplastic crises. A bar chart illustrating the frequency of presenting symptoms in patients with transient aplastic crisis. Values represent the proportion of patients presenting with each symptom

TAC presented predominantly with fever (93.4%), followed by nausea and vomiting (45.9%), weakness and fatigue (27.9%), headache (21.3%), abdominal pain and decreased appetite (19.7%). Cough (13.1%), diarrhea (8.2%), and rhinorrhea (6.6%) were less common. Rare manifestations included sore throat and skin rash, in one (1.6%) patient each (Fig. 1).

### Hematologic features of TAC

TAC episodes were characterized by marked cytopenias at the hematologic nadir. Severe anemia presented in all the patients. The mean hemoglobin level was 5.6 ± 1.0g/dL, compared with 9.9 ± 1.3g/dL at baseline. The mean reticulocyte percentage was 0.4 ± 0.2% versus 8.0 ± 3.1% at baseline, and the mean ARC at nadir was 11.1 ± 5.3 × 10^3^/µL. All the patients demonstrated reticulocyte percentage < 1%. The median WBC count at nadir was 3.2 × 10^3^/µL (IQR 2.3–4.8), and 63.6% (35/55) had leukopenia. The median ANC was 1.4 × 10^3^/µL (IQR 0.7–2.3), and 61.8% (34/55) had values below the age specific lower limit (1.0 × 10^3^/µL before age 1 year; l.5 × 10^3^/µL after age 1). The median platelet count was 122.5 × 10^3^/µL (IQR 85.8–185); thrombocytopenia presented in 65.5% (36/55) (Table 2). A median of 2 (IQR 1–2) RBC transfusions was required during the crisis.

**Table 2:** Clinical and hematologic characteristics of episodes of transient aplastic crisis

	Value
Age at crisis, years, median (IQR)	6.6 (4.6–8.2)
Laboratory values at nadir	
WBC (× 10^3^/µL), median (IQR)	3.2 (2.3–4.8)
ANC (× 10^3^/µL), median (IQR)	1.4 (0.7–2.3)
Hemoglobin (g/dL), mean ± SD	5.6 ± 1
Reticulocytes (%), mean ± SD	0.4 ± 0.2
ARC (× 10^3^/µL), mean ± SD	11.1 ± 5.3
Platelets (× 10^3^/µL), median (IQR)	122.5 (85.8–185)
Laboratory values at discharge	
WBC (× 10^3^/µL), median (IQR)	5.6 (3.8–8.1)
ANC (× 10^3^/µL), median (IQR)	2.2 (1.1–4.5)
Hemoglobin (g/dL), mean ± SD	9.4 ± 1.3
Reticulocytes (%), median (IQR)	0.5 (0.3–0.7)
ARC (× 10^3^/µL), median (IQR)	18 (11–30)
Platelets (× 10^3^/µL), median (IQR)	184 (124–298)
Parvovirus B19 testing (n)	
Positive	51/61 (83.6%)
Negative by serology ± PCR	3/61 (4.9%)
Not tested	7/61 (11.5%)
RBC transfusions, median (IQR)	2 (1–2)

*PCR* polymerase chain reaction, *WBC* white blood cell count, *ANC* absolute neutrophil count, *RBC* red blood cells, *SD* standard deviation, *IQR* interquartile range

Of 61 patients with TAC, 54 were tested for parvovirus-B19. Of those, 51 (94.4%) had positive parvovirus-B19 IgM or PCR. Two patients had negative IgM, and a PCR test was not performed. One patient had influenza-A virus during the aplastic crisis, with negative IgM and PCR tests for parvovirus-B19. Seven patients were not tested.

At hospital discharge, the mean hemoglobin level was 9.4 ± 1.3g/dL; anemia presented in 49/54 (90.7%). The median reticulocyte percentage was 0.5% (IQR 0.3–0.7); reticulocyte percentage < 1% persisted in 42/52 (80.8%). The median ARC at discharge was 18 × 10^3^/µL (IQR 11–30). The median WBC count increased to 5.6 × 10^3^/µL (IQR 3.8–8.1); 15/54 (27.8%) had values below the lower limit. The median ANC was 2.2 × 10^3^/µL (IQR 1.1–4.5); 16/54 (29.6%) had values below the age-specific lower limit. The median platelet count was 184 × 10^3^/µL (IQR 124–298; 21/54 (38.9%) had values below the lower limit) (Table 2).

### Risk factors for TAC

No statistically significant differences were observed between patients with and without documented TAC, with respect to age at diagnosis (1.8 ± 2.61 vs 1.5 ± 2.91 years, *p* = 0.468), baseline hemoglobin levels (9.93 ± 1.32 vs. 9.71 ± 1.49 g/dL, *p* = 0.388), baseline reticulocyte percentages (8% ± 3.06 vs 7.79% ± 2.93, *p* = 0.699), cholecystectomy (42.62% vs. 40.32%, *p* = 0.796), the number of hospitalizations (1.97 ± 2.86 vs. 2.52 ± 3.23, *p* = 0.319) or the number of RBC transfusions (3.75 ± 7.18 vs 5.66 + 8.64, *p* = 0.185).

### TAC-related pancytopenia

We performed a comprehensive comparison of disease characteristics between patients who developed pancytopenia and those who did not. Of the 61 patients who experienced TAC, complete blood count data at admission were available for 55, of whom 28 (50.9%) developed pancytopenia. Bipenia occurred in an additional 12 (21.8%): 6 (10.9%) had anemia and leukopenia and 6 (10.9%) had anemia and thrombocytopenia. The median age of the patients with pancytopenia was older at the crisis (6.7 years, IQR 6–8.4 vs. 5.4, IQR 3.7–7.8, *p* = 0.033). Other clinical characteristics were comparable between patients with and without pancytopenia, with no statistically significant differences across symptoms (fever, nausea and vomiting, weakness and fatigue, and headache) at presentation. Fever was the most frequent symptom in patients with and without pancytopenia (27 (96.4%) vs. 24 (88.9%), *p* = 0.352).

The mean hemoglobin level at nadir was lower in the pancytopenia group (5.3 ± 1.0 vs. 5.9 ± 0.8g/dL, *p* = 0.009). In agreement, patients with pancytopenia required significantly more RBC transfusions during TAC (median 2, IQR 1.8–2 vs. 1, IQR 1–2) units, *p* < 0.001). The mean reticulocyte percentage did not differ significantly between the groups (0.36 ± 0.19% vs. 0.39 ± 0.17%, *p* = 0.540).

At discharge, thrombocytopenia persisted in the pancytopenia group, affecting 20 of 28 patients (71.4%). The mean time to resolution of pancytopenia was 7.2 ± 3.3 days.

### Baseline HS severity and the clinical course of TAC

To evaluate the relation between baseline HS severity and the clinical course of TAC, we analyzed the baseline hemoglobin level, the baseline reticulocyte percentage, and the prior transfusion burden, in relation to transfusion requirements during TAC and the occurrence and severity of pancytopenia.

#### Transfusion requirements during TAC

**Fig. 2:**
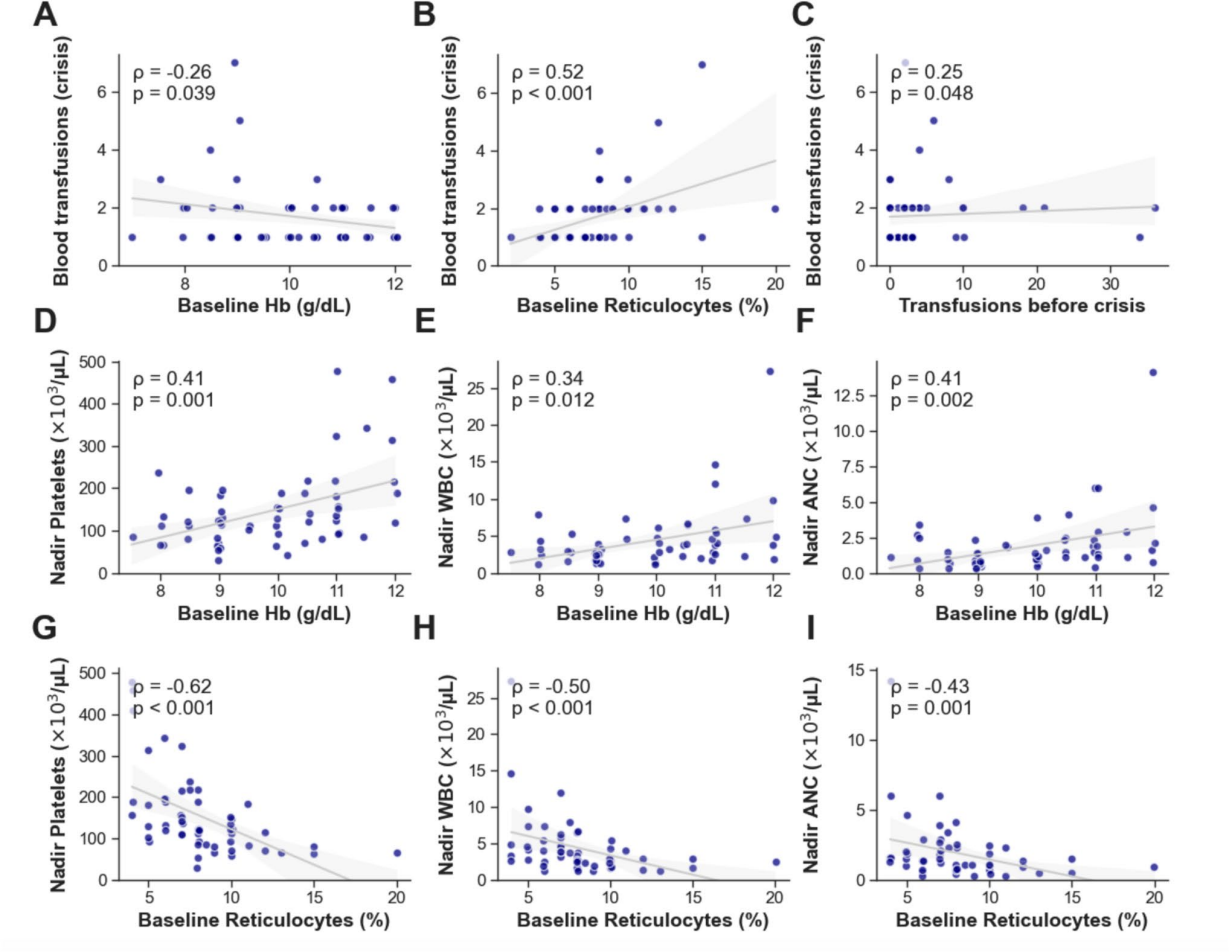
Correlations between baseline hematologic indices and the severity of transient aplastic crisis. Each dot represents a single patient, points are jittered to reveal overlapping data. Spearman correlation coefficients (ρ) and *p*-values are shown. (**A**) correlation between baseline hemoglobin (g/dL) and blood transfusions during crisis, (**B**) correlation between baseline reticulocytes (%) and blood transfusions during crisis, (**C**) correlation between blood transfusions before crisis and blood transfusions during crisis, (**D**) correlation between baseline hemoglobin (g/dL) and nadir platelet counts (× 10^3^/µL), (**E**) correlation between baseline hemoglobin (g/dL) and nadir white blood cell counts (× 10^3^/µL), (**F**) correlation between baseline hemoglobin (g/dL) and nadir absolute neutrophil counts (× 10^3^/µL), (**G**) correlation between baseline reticulocytes (%) and nadir platelet counts (× 10^3^/µL), (**H**) correlation between baseline reticulocytes (%) and nadir white blood cells counts (× 10^3^/µL), (**I**) correlation between baseline reticulocytes (%) and nadir absolute neutrophil counts (× 10^3^/µL). Hb, hemoglobin

Lower baseline hemoglobin levels were modestly but significantly associated with increased transfusion requirements during TAC (Spearman’s *ρ* = − 0.26, *p* = 0.039, Fig. 2A). Higher baseline reticulocyte percentage demonstrated a strong positive correlation with the number of transfusions required during the crisis (*ρ* = 0.52, *p* < 0.001, Fig. 2B). Similarly, a greater prior transfusion burden was associated with increased RBC transfusion requirements during TAC, although the strength of this association was weaker (*ρ* = 0.25, *p* = 0.048, Fig. 2C).

#### Pancytopenia during TAC

Among patients who developed pancytopenia during TAC, compared to those who did not, the mean baseline hemoglobin level was lower (9.6 ± 1.2 vs. 10.3 ± 1.3, *p* = 0.026) and the median baseline reticulocyte percentage was higher (9.0%, IQR 8.0–10.0 vs. 7.0%, IQR 5.0–7.5; Mann–Whitney *U* = 615.0, *p* < 0.001). The median prior transfusion burden did not differ significantly between those with and without pancytopenia (2.0, IQR 0–5.3 vs. 1.0 IQR 0–3; Mann–Whitney U = 469.5, *p* = 0.112) (Table 3).

**Table 3:** Risk factors of pancytopenia during transient aplastic crisis

	Pancytopenia	No pancytopenia	*p*-value
Baseline hemoglobin (g/dL), mean ± SD	9.6 ± 1.2	10.3 ± 1.3	0.026
Baseline reticulocyte percentage, median (IQR)	9.0 (8.0–10.0)	7.0 (5.0–7.5)	< 0.001
Prior transfusion burden, median (IQR)	2.0 (0.0–5.3)	1.0 (0.0–3.0)	0.112

Baseline hemoglobin demonstrated a moderate positive correlation with nadir platelet counts (*ρ* = 0.41, *p* = 0.001, Fig. 2D), a weak-moderate positive correlation with nadir WBC counts (*ρ* = 0.34, *p* = 0.012, Fig. 2E), and a moderate positive correlation with nadir ANC (*ρ* = 0.41, *p* = 0.002, Fig. 2F).

Baseline reticulocyte percentages demonstrated a strong negative correlation with nadir platelet counts (*ρ* = −0.62, *p* < 0.001, Fig. 2G), a moderate negative correlation with nadir WBC counts (*ρ* = −0.50, *p* < 0.001, Fig. 2H), and a moderate negative correlation with nadir ANC (*ρ* = −0.43, *p* = 0.001, Fig. 2I). No significant correlation was found between baseline reticulocyte percentages and nadir hemoglobin levels during TAC (*ρ* = − 0.165, *p* = 0.224).

#### Multivariable analysis

**Fig. 3:**
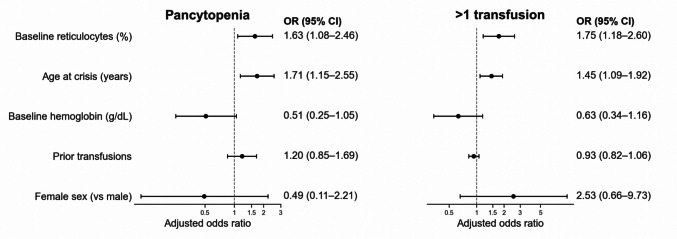
Forest plots of adjusted odds ratios (OR) for pancytopenia (left) and the requirement of > 1 transfusion (right) during a transient aplastic crisis. The points represent adjusted ORs and the horizontal lines indicate 95% confidence intervals derived from multivariable logistic regression models. The dashed vertical line denotes the null value (OR = 1). The x-axis is displayed on a logarithmic scale. Predictor variables are listed on the y-axis and are identical across both panels. OR, odds ratio; CI, confidence interval

In a multivariable logistic regression analysis, we examined the risk of developing pancytopenia during TAC. The model included baseline reticulocyte percentage, baseline hemoglobin, prior transfusion burden, age at the crisis, and patient sex. Baseline reticulocyte percentage and age at the crisis were independently associated with pancytopenia (Fig. 3). Specifically, each 1% increase in baseline reticulocyte percentage was associated with a 63% increase in the odds of pancytopenia (adjusted odds ratio (OR) 1.63, 95% confidence interval (CI) 1.08–2.46; *p* = 0.019). Each additional year of age at the crisis was associated with a 71% increase in the odds of pancytopenia (adjusted OR 1.71, 95% CI 1.15–2.55; *p* = 0.009). Baseline hemoglobin demonstrated a borderline inverse association with the risk of pancytopenia (adjusted OR 0.51, 95% CI 0.25–1.05; *p* = 0.068).

In a multivariable model predicting transfusion burden during TAC, higher baseline reticulocyte percentage and older age at the crisis remained independent predictors of a requirement of > 1 RBC transfusion (Fig. 3). Specifically, each 1% increase in baseline reticulocyte percentage was associated with a 75% increase in the odds of requiring > 1 transfusion (adjusted OR 1.75, 95% CI 1.18–2.60; *p* = 0.005), and each additional year of age at the crisis was associated with a 45% increase in the odds of requiring > 1 transfusions (adjusted OR 1.45, 95% CI 1.09–1.92; *p* = 0.010). In contrast, baseline hemoglobin, prior transfusion burden, and patient sex were not independently associated with this outcome. No significant interaction between baseline hemoglobin and baseline reticulocyte percentage was observed in either model.

## Discussion

In this large pediatric cohort of patients with HS, we demonstrated that TAC, a common complication of HS, often manifests with multilineage cytopenias. Importantly, we found that baseline markers of HS severity, including higher reticulocyte percentage, lower hemoglobin levels and greater transfusion requirements prior to TAC, were associated with increased TAC severity.

HS severity is commonly assessed according to hemoglobin levels and reticulocyte counts [7]. In 60–70% of patients, hemoglobin levels have been reported in the range of 8–12 g/dL and reticulocyte percentage in the range of 6–10% [3, 7]. Overall, the clinical and laboratory features of our cohort fall within these ranges and are consistent with the severity distribution described in the literature [3, 7, 27].

About half our patients experienced TAC. This compares to poorly defined prevalences in the literature [7, 13]. Eldbary et al. prospectively followed 75 patients with HS, of whom 19 (25.3%) developed symptomatic parvovirus-B19 infection [17]. The median age of our affected patients was 6 years, consistent with the known predilection of parvovirus-B19 infection for children aged 4–12 years [28]. Our lack of identification of statistically significant baseline risk factors for the development of TAC suggests unpredictable occurrence in patients with HS.

Overall, 83.6% of our patients with TAC had evidence of acute parvovirus-B19 infection, and among those tested, 94.4% had a proven infection. One patient with TAC had influenza-A infection, an association rarely reported [7]. Current guidelines recommend a combined diagnostic approach for parvovirus-B19, using both serologic testing and PCR [13, 14, 29]. Most of our patients were evaluated by serology. Notably, none had a positive PCR result in the absence of serologic evidence of infection. While these observations should be interpreted cautiously given the limited use of PCR, they suggest that serologic testing alone may be a reliable diagnostic tool for acute parvovirus-B19 infection in this setting.

We found that the most common presenting symptoms of TAC were fever and gastrointestinal complaints, whereas the characteristic rash of parvovirus-B19 infection was uncommon, consistent with previous reports [7, 18]. Hepatic and renal involvement were not identified in our study. In contrast, in a prospective study, patients with HS and parvovirus-B19 infection frequently presented with marked direct hyperbilirubinemia, acute hepatitis and acute kidney injury [17].

Although TAC is traditionally described with pure red cell aplasia, evidence suggests that parvovirus-B19 infection can be associated with multi-lineage cytopenias. Prior case reports and small series have documented pancytopenia during parvovirus-B19-associated TAC in patients with HS and other hemolytic anemias [12, 18–21]. Pancytopenia was observed in about half of our patients with TAC. The pathophysiology of pancytopenia is likely multifactorial, involving both direct viral toxicity and immune-mediated mechanisms. Direct effects arise from viral tropism for erythroid and non-erythroid progenitors via the P-antigen [13]. Concurrently, acute infection triggers robust immune activation with elevated pro-inflammatory cytokines that suppress multilineage hematopoiesis [18]. Immune-mediated peripheral platelet destruction may further contribute to thrombocytopenia [13, 30].

Several reports suggested that the severity of the underlying hemolytic disorder may influence the clinical presentation of TAC, although this relation has not been systematically examined [31, 32]. In univariate analyses, we found that lower baseline hemoglobin and higher baseline reticulocyte percentage were associated with increased TAC severity, manifested by higher rates of pancytopenia and greater transfusion requirements during crisis. In multivariable models, baseline reticulocyte percentage remained independently associated with TAC severity, and particularly with a requirement for multiple transfusions. This supports the concept that patients with sustained high erythropoietic activity at baseline are more susceptible to abrupt interruption of RBC production. In addition, patients with pancytopenia required substantially more RBC transfusions during TAC. These findings suggest that patients with more severe baseline characteristics and those presenting with pancytopenia require close monitoring of their hemoglobin levels during and following TAC.

Older age at presentation was also independently associated with both pancytopenia and increased transfusion requirements during TAC. As primary parvovirus-B19 infection is often reported as clinically more symptomatic in younger children [28], the mechanisms underlying this age-related association in HS remain unclear and warrant further investigation. Rather than a different disease mechanism, the association between older age and increased transfusion requirement likely reflects, at least in part, higher body weight and blood volume, necessitating larger transfusion volumes to correct anemia during crisis. In addition, better hemodynamic tolerance for anemia in younger patients may have contributed to this finding.

All our patients, including those with more severe hematologic involvement, experienced complete recovery. None developed sustained BM failure, and BM was therefore not examined. TAC resolved after a mean of 7.2 ± 3.3 days, which is shorter than the 10–14 days typically reported in the literature [3, 7]. This difference may partly reflect our calculation of resolution from the first day of hospitalization rather than from symptom onset.

Several limitations of this study should be acknowledged. Its retrospective design and extended study period may have introduced variability in diagnostic and treatment approaches. Specifically, during the study period, parvovirus-B19 testing modalities changed. In addition, baseline hematologic parameters were derived from available pre-crisis measurements and may have been influenced by intercurrent illness or recent transfusions. Despite these limitations, the relatively large cohort and the consistency of associations observed across analyses support the robustness and clinical relevance of our findings.

### Conclusions

While the occurrence of TAC in HS is unpredictable, its clinical course is closely linked to the severity of the underlying hemolytic disease. Baseline HS severity, characterized by lower hemoglobin levels and elevated reticulocyte percentage, was independently associated with more severe TAC manifestations, including pancytopenia and increased transfusion requirements. In addition, TAC-related pancytopenia was associated with the requirement of more than one transfusion. These findings suggest that patients with a severe HS phenotype may benefit from closer monitoring during TAC, considering the potential need for repeated transfusions. Overall, incorporating baseline disease severity into the clinical assessment may improve risk stratification and guide supportive management during TAC in patients with HS.

## Data Availability

No datasets were generated or analysed during the current study.

## Abbreviations

ANC: Absolute neutrophil count
ARC: Absolute reticulocyte count
BM: Bone marrow
CI: Confidence interval
DNA: Deoxyribonucleic acid
ELISA: Enzyme-linked immunosorbent assay
EMA: Eosin-5-maleimide
HS: Hereditary spherocytosis
IgM: Immunoglobulin M
IQR: Interquartile range
NS-1: Non-structural protein 1
OR: Odds ratio
PBS: Phosphate-buffered saline
PCR: Polymerase chain reaction
RBC: Red blood cell
SD: Standard deviation
TAC: Transient aplastic crisis
WBC: White blood cell
